# Diplopia and Ptosis: An Unusual Case of Prostate Cancer Metastasis to the Sphenoid Bone Treated With Palliative Radiotherapy

**DOI:** 10.7759/cureus.50566

**Published:** 2023-12-15

**Authors:** Mahvish Renzu, Ishaan J Bhatt, Saad Ahmed, Akhil Jain, Oleg M Teytelboym, Gregory C Stachelek, Rajesh Thirumaran

**Affiliations:** 1 Internal Medicine, Trinity Health Oakland, Wayne State University Program, Pontiac, USA; 2 Internal Medicine, Drexel University College of Medicine, Philadelphia, USA; 3 Internal Medicine, Mercy Catholic Medical Center, Darby, USA; 4 Radiology, Mercy Catholic Medical Center, Darby, USA; 5 Radiation Oncology, Mercy Catholic Medical Center, Darby, USA; 6 Hematology and Oncology, Mercy Catholic Medical Center, Darby, USA

**Keywords:** radiotherapy (rt), radiotherapy, chemotherapy, neuroendocrine carcinoma of prostate, adenocarcinoma prostrate cancer, diplopia, prostrate cancer

## Abstract

We report a case of a 72-year-old male who presented to the hospital with a chief complaint of diplopia in the setting of a recent onset of urinary incontinence and right-sided back pain. He was subsequently diagnosed with prostate cancer, notably metastasizing to the right sphenoid bone, causing impingement of the oculomotor nerve. Our case is unique in that the patient’s initial presentation of prostate cancer was oculomotor nerve palsy with subsequent histologic analysis of the primary tumor showing both small cell neuroendocrine carcinoma along with adenocarcinoma. Also, the initial routine stroke protocol MRI and computed tomography angiography (CTA) missed the lesion, while gadolinium-enhanced targeted MRI revealed lesions in both the spine and the orbit. This case emphasizes the need for enhanced contrast as well as focused imaging in patients presenting with diplopia with a negative initial workup for stroke. Ptosis can be a sign of metastasis from other cancers and it is important to have a broad differential including metastatic disease in patients' presenting with similar symptoms and negative initial workup who may otherwise be at risk of cancer.

## Introduction

Prostate cancer is the most common malignancy among men and is the second-leading cause of cancer death in men [1]. In contrast to other cancers that commonly spread to brain, it is unusual for prostate carcinoma to metastasize to the central nervous system (CNS), making it the subject of case reports due to the uncommon event of neurologic side effects.

We report a case of a 72-year-old male who presented to the hospital with a chief complaint of diplopia in the setting of a recent onset of urinary incontinence and right-sided back pain and was subsequently diagnosed with prostate cancer, notably metastasizing to the right sphenoid bone, causing impingement of the oculomotor nerve. Unlike previously few reported cases of oculomotor nerve palsy due to prostate cancer with non-adenocarcinoma pathology, our case biopsy uncovered neuroendocrine and adenocarcinoma histology, which is a rare phenomenon, and the patient received palliative orbital radiotherapy.

This case report highlights the significance of employing a multidisciplinary diagnostic approach and emphasizes the pivotal role of palliative radiotherapy in alleviating symptoms related to rare skeletal metastases. Additionally, it underscores the significance of utilizing advanced imaging techniques for the early detection of such rare instances. Increased awareness of these atypical manifestations can contribute to prompt intervention and enhance outcomes in similar cases.

## Case presentation

A 72-year-old male with medical history of hypertension, hyperlipidemia, prediabetes, Lyme disease, coronary artery disease, hepatitis C, 50-pack-year smoking history, and diverticulitis was admitted for four days of worsening diplopia and right-sided ptosis. His physical examination revealed ptosis with asymmetric pupils (right 2.5 mm, left 3 mm), bilaterally reactive to light and accommodation. The right eye was depressed and abducted with a disconjugate gaze. On rightward gaze, diplopia improved but on leftward gaze, the right eye did not adduct, diplopia worsened, and nystagmus was noted. Cranial nerves I, II, and IV-XII were intact, and no other neurological deficits were observed. Initial lab workup was unremarkable in explaining his symptoms. 

**Figure 1:**
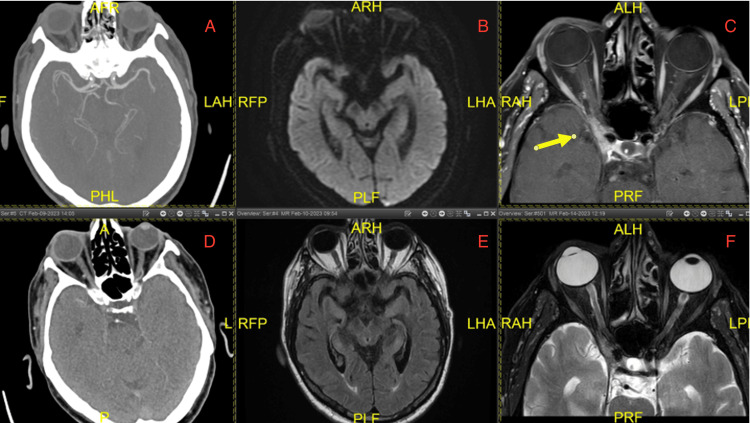
CTA and MRI of head and neck. (A) and (B): Computed tomography angiography (CTA) head and neck without any large vessel occlusion. (C) and (D): MRI brain with stroke protocol DWI and Flair without any evidence of stroke or metastasis. (E) and (F): T1 and T2 images of dedicated MRI orbits with contrast showing metastasis in the right orbit and cavernous sinus.

Workup for stroke, including CT angiography of the head and neck (Figures 1A, 1B) and stroke protocol MRI brain without contrast (Figures 1C, 1D), were essentially within normal limits. Autoimmune workup revealed antinuclear antibody (ANA) titer 1:640 but negative for anti-SCL-70, anti-Smith, anti-dsDNA, anti-Ro/SSA and anti-La/SSB, c-ANCA, p-ANCA, rheumatoid factor, anti-sm/RNP, and acetylcholine receptor-blocking antibodies. The viral hepatitis panel was positive for hepatitis C antibodies but undetectable for hepatitis C virus (HCV) RNA. A CT scan of the chest with contrast showed bilateral subtle pulmonary nodules (the largest being 6 mm in the right upper lobe).

**Figure 2:**
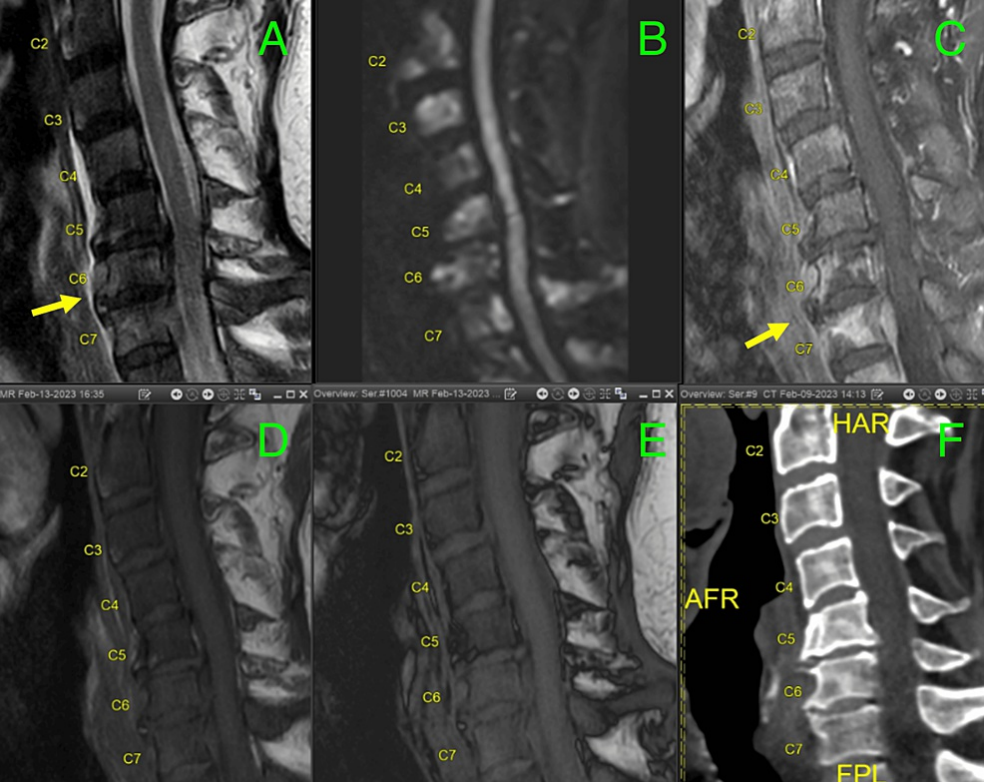
MRI cervical spine. (A) and (B) MRI cervical spine T1 and T2 with an abnormal marrow signal. (C) and (D) Diffusion-weighted signal imaging of (C) spine showing extensive signals from vertebral bodies suspicious of metastasis. (E) and (F) Enhancement on the vertebral body of C7 with enhancement behind it representing epidural spread and displacement of cord similar to seen on (A).

Later, during his hospital stay, he complained of severe bilateral back pain, more in the mid-scapular region. He endorsed having had similar complaints intermittently for the last two weeks. MRI of the cervical spine (Figure 2) revealed multiple vertebral lesions and abnormal marrow signals concerning diffuse metastatic disease. On detailed review, he endorsed worsening urinary urgency, hesitancy, incomplete emptying, and nocturia despite taking tamsulosin for the last three months. He was supposed to follow up with the urologist for the urinary symptoms and check prostate-specific antigen (PSA) levels, but unfortunately, due to some reasons, it was delayed. His serum PSA levels during the hospitalization were found to be significantly elevated (226.3 ng/ml).

Contrast MRI of the orbits revealed right sphenoid metastasis with extraosseous extension into the right cavernous sinus, causing compression of the third cranial nerve. CT abdomen and pelvis with contrast showed right prostate apex mass with lymph node involvement, liver metastasis, as well as extensive skeletal metastasis.

**Figure 3:**
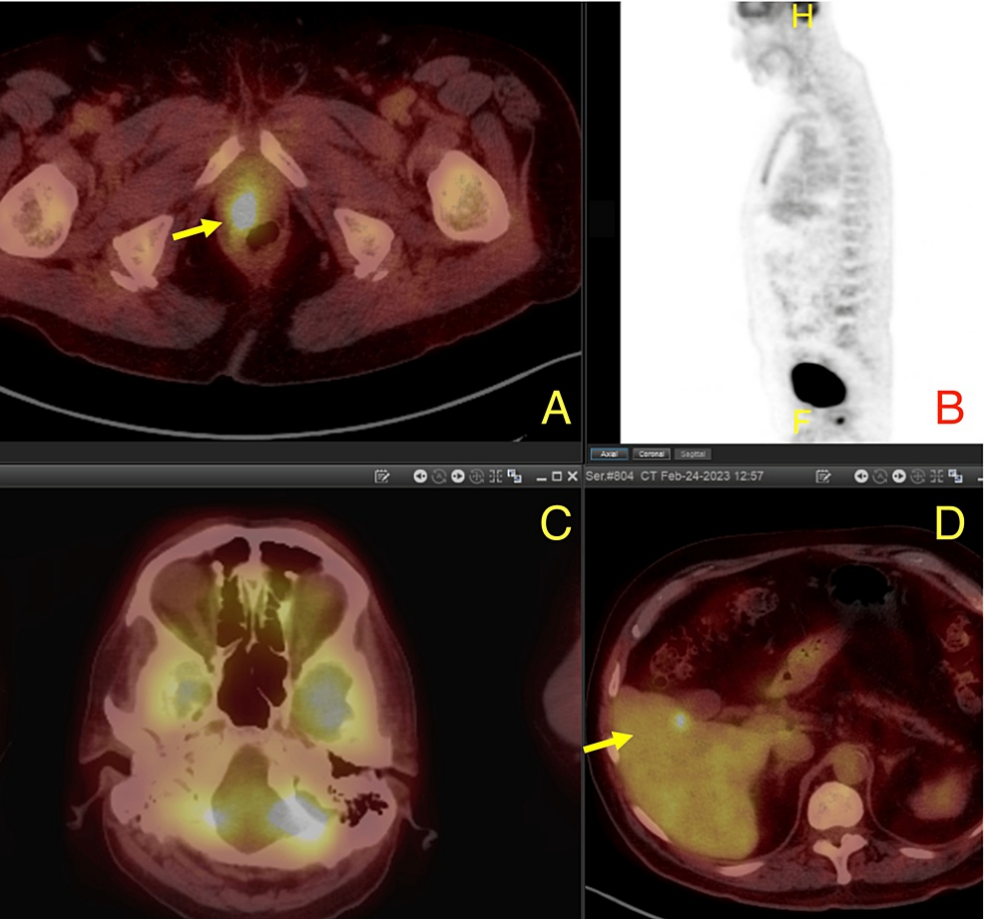
FDG-PET CT. (A) Increased uptake seen in apical-lateral prostate similar to area showing small cell neuroendocrine growth. (B) Skull not demonstrating enhanced growth near the area of orbital metastasis. (C) Spine not showing significant FDG enhancement. (D) Liver metastasis demonstrating increased uptake to FDG. FDG-PET: 18 F-fluorodeoxyglucose-positron emission tomography.

He underwent ultrasound-guided prostatic biopsy, which showed small cell neuroendocrine carcinoma (3/12 cores) and adenocarcinoma (Gleason 7 8/12 cores, Gleason 9 1/12 cores). Immunohistochemistry was positive for TTF-1, synaptophysin, and chromogranin. 18 F-fluorodeoxyglucose-positron emission tomography (FDG-PET) showed increased FDG uptake in the apical and lateral areas, consistent with the area showing a small cell neuroendocrine tumor on prostate biopsy, while adenocarcinoma, which was seen to be extensively present throughout the prostate, did not demonstrate increased FDG uptake (Figure 3A). Areas of orbital as well as epidural and vertebral metastasis did not demonstrate increased FDG uptake (Figures 3B, 3C), while areas of liver metastasis did demonstrate increased FDG uptake (Figure 3D).

He was treated with dexamethasone and urgent palliative radiotherapy to the skull base (3000 cGy in 10 fractions) and C5-T1 (2000 cGy in five fractions) using a 3D conformal approach. His acute complaint of diplopia was eventually alleviated. He followed up with medical oncology outpatient, he was initiated on chemotherapy along with immunotherapy, which included carboplatin intravenous (I.V.) infusion on day 1, etoposide I.V. infusion on days one, two, and three, and atezolizumab I.V. infusion on day one, followed by maintenance therapy with atezolizumab I.V. infusion once every 21 days. He was also started on darolutamide (anti-androgen medication) 300 mg twice a day daily along with Lupron (hormone-modulating drug) every three months. Later on, lurbinectedin 3.2 mg (an alkylating agent) every three weeks was added to the chemotherapy regimen. In view of preventing skeletal-related events, he was started on bone anti-resorptive therapy called Xgeva and Percocet for pain. The patient's serum creatinine returned to baseline, and PSA decreased to 6.87 ng/mL. Re-staging CT after a couple of months showed a treatment response. He also received intermittent palliative radiation therapy, which considerably improved his pain, and he has been ambulating more steadily.

## Discussion

Prostate cancer is the second most common malignancy among men, with a range of clinical presentations, often urinary symptoms or bone pain due to metastasis [2]. Diagnostic confirmation involves prostate biopsy displaying adenocarcinoma, small cell carcinoma, and/or neuroendocrine phenotypes with grading based on the Gleason scoring system [3]. Metastasis is usually to the bone (84%), and much less commonly to distant lymph nodes (10.6%), liver (10.2%), thorax (9.1%), and brain (3.1%) [4].

Our case is unique in that the patient’s initial presentation of prostate cancer was oculomotor nerve palsy with subsequent histologic analysis of the primary tumor showing both small cell neuroendocrine carcinoma along with adenocarcinoma with intermediate-to-high risk Gleason scores. Although intracranial metastasis from prostate adenocarcinoma is extremely rare, brain metastasis from other types of prostate tumors is much higher; however, in our case, FDG-PET shows that this is likely from adenocarcinoma and not from neuroendocrine prostate cancer (NEPC)/small cell. The utility of FDG-PET in identifying brain metastases has often been scrutinized in the literature, revealing limitations due to its low sensitivity. According to some reports, PET could only detect 61-68% of metastatic lesions compared to those identified by MRI. This highlights the superior diagnostic capability of MRI in the context of brain metastasis detection, especially in adenocarcinomas [5]. Prostate adenocarcinoma is characterized by morphological features resembling luminal prostate cells, is androgen-driven, and is typically associated with elevated serum prostate-specific antigen (PSA). On the other hand, neuroendocrine prostate cancer (NEPC) is an aggressive subtype that can emerge spontaneously or develop in advanced stages of prostate cancer, or often as a result of treatment resistance. Patients with pathologically confirmed NEPC commonly exhibit visceral metastases, low PSA levels, and frequent loss of the RB1 and TP53 genes [6].

Two previous case reports have reported oculomotor nerve palsies as the presenting symptom of prostate cancer, but neither describes adenocarcinoma histology or the use of palliative radiotherapy [7,8]. Among 27 previously diagnosed prostate cancer patients who received bone scintigraphy, only 1 (3.1%) had skull metastasis [9]. A recent retrospective study reported that patients who had skull metastases had significantly higher biopsy Gleason scores, higher clinical T-stage, and shorter overall survival [10]. While the patient in this case did not have brain metastasis, it has been noted that the tumor histology impacts the likelihood of brain metastasis with small cell and primary transitional cell carcinomas more likely to do so than adenocarcinoma [11]. Also, prostate cancer with brain metastasis often involves mutations of homologous recombination repair genes, including BRCA1, BRCA2, and many others [12]. Though cerebrovascular accident (CVA), trauma, myasthenia gravis, granulomatous lesions, and multiple sclerosis were differential diagnoses for his diplopia, the patient’s age, urinary symptoms, musculoskeletal pain, and initial diagnostic workup prompted further evaluation of prostate cancer. This patient’s imaging showed compressive effects on the oculomotor nerve at the right sphenoid bone. In addition, palliative radiation therapy has been successful in alleviating the immediate symptoms.

Since 1940's androgen deprivation therapy (ADT) alone has been the standard of care for many years in men with metastatic prostate cancer. Due to the limited survival under this monotherapy, many new treatment options have been developed in recent years. Especially for hormone-sensitive prostate cancer, combination therapies of two or three agents of ADT, androgen receptor signaling inhibitors (ARSIs), and chemotherapy have proven effective, resulting in a substantial improvement in overall survival [13]. The latest findings from cohort 6 in the COSMIC-021 study have reignited enthusiasm in the field of immunotherapy for metastatic prostate cancer. The study revealed a notable 32% response rate and an impressive 80% disease control rate when utilizing the combination of chemotherapy and immunotherapy. These promising results underscore the potential of immunotherapeutic in addressing this complicated disease [14]. GnRH agonists such as leuprolide and anti-androgens such as bicalutamide can be used [15]. Chemotherapy regimens often include docetaxel, cabazitaxel, and corticosteroid [16]. Palliative radiotherapy offers a speedy, economical, and compelling approach to decreasing large numbers of focal symptoms, especially in advanced cancer. Numerous studies so far have proven significant pain control and improvement in quality of life in about 50-60% of patients after receiving palliative radiotherapy, like in our case [17]. Typically, adenocarcinoma is androgen-sensitive and treated by ADT, while NEPC is rare, not androgen- or hormone-sensitive, more aggressive, and treated by chemotherapy. As mentioned earlier in neuroendocrine tumors, PSA levels are low due to a lack of expression of androgen receptors; hence, serum PSA does not correlate with disease burden. In our case, elevated PSA gave a clear diagnostic cue towards prostate as a sight of primary; however, this correlation is more prominent in patients with adenocarcinoma and not NEPC. In the event that the patient with isolated NEPC with metastasis, further testing and workup will be required.

The attribution of prostate cancer metastasis as the cause of this patient’s oculomotor nerve palsy occurred after extensive evaluation for other causes, and imaging of the pelvis was only completed after realizing the patient had cervical spine metastasis. It is also interesting to note that despite the presence of seemingly aggressive disease from NEPC, which had metastasized to the bone, our patient’s symptoms were discovered through metastasis from relatively less aggressive adenocarcinoma, which was widespread.

## Conclusions

Ptosis itself is a rare presenting feature of prostate cancer. Other case reports describe ptosis as a presentation, but they do not describe the neuroendocrine histology. In addition, routine stroke protocol MRI and CTA missed the lesion, while gadolinium-enhanced targeted MRI revealed lesions in both the spine and the orbit. This case emphasizes the need for contrast-enhanced, as well as focused imaging in patients presenting with diplopia with negative initial workup for stroke. Ptosis can be a sign of metastasis from other cancers, including breast cancer, head and neck cancer, thyroid cancer, lymphoid or neuroblastoma, and it is important to have a broad differential including metastatic disease in patients presenting with similar symptoms and negative workup who may otherwise be at risk of cancer. The case illustrates the importance of physical examination, diagnostic evaluation, and radiologic imaging in providing appropriate care to a patient. 

As a final note, this case report suggests that a more extensive study encompassing more patient samples could further enhance our understanding of the clinical nuances associated with ptosis as a presentation of prostate cancer. Such studies would aid in refining diagnostic and therapeutic strategies for similar presentations in the future.
